# Heart Rate Information-Based Machine Learning Prediction of Emotions Among Pregnant Women

**DOI:** 10.3389/fpsyt.2021.799029

**Published:** 2022-01-27

**Authors:** Xue Li, Chiaki Ono, Noriko Warita, Tomoka Shoji, Takashi Nakagawa, Hitomi Usukura, Zhiqian Yu, Yuta Takahashi, Kei Ichiji, Norihiro Sugita, Natsuko Kobayashi, Saya Kikuchi, Yasuto Kunii, Keiko Murakami, Mami Ishikuro, Taku Obara, Tomohiro Nakamura, Fuji Nagami, Takako Takai, Soichi Ogishima, Junichi Sugawara, Tetsuro Hoshiai, Masatoshi Saito, Gen Tamiya, Nobuo Fuse, Shinichi Kuriyama, Masayuki Yamamoto, Nobuo Yaegashi, Noriyasu Homma, Hiroaki Tomita

**Affiliations:** ^1^Department of Psychiatry, Tohoku University Graduate School of Medicine, Sendai, Japan; ^2^Department of Psychiatry, Tohoku University Hospital, Sendai, Japan; ^3^Department of Preventive Medicine and Epidemiology, Tohoku University Tohoku Medical Megabank Organization, Sendai, Japan; ^4^Department of Disaster Psychiatry, Tohoku University International Research Institute of Disaster Sciences, Sendai, Japan; ^5^Department of Radiological Imaging and Informatics, Tohoku University Graduate School of Medicine, Sendai, Japan; ^6^Department of Management, Science and Technology, Graduate School of Engineering, Tohoku University, Sendai, Japan; ^7^Department of Health Record Informatics, Tohoku University International Research Institute of Disaster Sciences, Sendai, Japan; ^8^Department of Public Relations and Planning, Tohoku University International Research Institute of Disaster Sciences, Sendai, Japan; ^9^Department of Community Medical Supports, Tohoku University Tohoku Medical Megabank Organization, Sendai, Japan; ^10^Department of Obstetrics, Tohoku University Graduate School of Medicine, Sendai, Japan; ^11^Department of Integrative Genomics, Tohoku University Tohoku Medical Megabank Organization, Sendai, Japan

**Keywords:** pregnancy, emotion, heart rate variability, autonomic system, machine learning, ensemble learning, random forest, gradient boosting trees

## Abstract

In this study, the extent to which different emotions of pregnant women can be predicted based on heart rate-relevant information as indicators of autonomic nervous system functioning was explored using various machine learning algorithms. Nine heart rate-relevant autonomic system indicators, including the coefficient of variation R-R interval (CVRR), standard deviation of all NN intervals (SDNN), and square root of the mean squared differences of successive NN intervals (RMSSD), were measured using a heart rate monitor (MyBeat) and four different emotions including “happy,” as a positive emotion and “anxiety,” “sad,” “frustrated,” as negative emotions were self-recorded on a smartphone application, during 1 week starting from 23rd to 32nd weeks of pregnancy from 85 pregnant women. The k-nearest neighbor (k-NN), support vector machine (SVM), logistic regression (LR), random forest (RF), naïve bayes (NB), decision tree (DT), gradient boosting trees (GBT), stochastic gradient descent (SGD), extreme gradient boosting (XGBoost), and artificial neural network (ANN) machine learning methods were applied to predict the four different emotions based on the heart rate-relevant information. To predict four different emotions, RF also showed a modest area under the receiver operating characteristic curve (AUC-ROC) of 0.70. CVRR, RMSSD, SDNN, high frequency (HF), and low frequency (LF) mostly contributed to the predictions. GBT displayed the second highest AUC (0.69). Comprehensive analyses revealed the benefits of the prediction accuracy of the RF and GBT methods and were beneficial to establish models to predict emotions based on autonomic nervous system indicators. The results implicated SDNN, RMSSD, CVRR, LF, and HF as important parameters for the predictions.

## Introduction

Heart rate variability (HRV) is often used to characterize the function of autonomic nervous system activity by analyzing time and frequency domains based on normal-to-normal (NN) intervals (1). Time domain features include the coefficient of variation R-R interval (CVRR), standard deviation of all NN intervals (SDNN), square root of the mean squared differences of successive NN intervals (RMSSD), the number of interval differences of successive RR-intervals >50 ms (NN50), and proportion derived by dividing NN50 by the total number of RR-intervals (pNN50). Frequency domain features include low frequency from 0.04 to 0.15 Hz (LF), high frequency from 0.15 to 0.4 Hz (HF), and the ratio of low frequency to high frequency (LF/HF).

HRV reflects many physiological and psychological factors, including emotions. Emotions affect our daily lives. However, emotions can also reflect mental conditions and significantly correlate with physical health (2). Negative emotions induce physiological arousal in a manner specific to the type of emotion (3). Physiological arousal can be measured as the change in HRV. Many researchers have focused on the effects of different emotions and HRV. For example, Xiu et al. (4) indicated that the HF reflects emotion and used HF to assess the effect of working memory training on emotion regulation. Rakshit et al. (5) used HRV features to classify different types of emotions, including happy and sad and neutral or null emotions. Goldstein et al. (6) suggested that HRV can be used as a marker to recognize different emotions.

Many researchers have combined AI to establish a prediction model to predict changes in emotion based on HRV. However, the previous studies applied only a limited number of machine learning algorithms among many widely used algorithms. For example, Li et al. (7) provided a comprehensive overview of physiological signal-based emotion recognition. In this study, the authors enumerated various studies on physiological signal types and various machine learning algorithms for emotion recognition. The diverse algorithms include support vector machine (SVM), k-nearest neighbor (k-NN), decision tree (DT), and random forest (RF). Among them, several studies addressed emotion classifiers (8–11). SVM has been implicated as an appropriate method to discriminate among different emotions (5, 12–15). Two studies reported that logistic regression (LR) is an appropriate method to distinguish emotions (16, 17). Two other studies demonstrated that k-NN can be used as an emotion classifier (18, 19). Naïve Bayes (NB) is a proper method to predict emotions (20–22). RF can solve the problem of emotion recognition with a higher accuracy than that of a few other methods (23–25). Lee et al. (26) utilized an artificial neural network (ANN) to distinguish different emotions. Besides the seven algorithms mentioned above, several new machine learning algorithms have been developed. These include stochastic gradient descent (SGD), gradient boosting trees (GBT), and extreme gradient boosting (XGBoost). The application of these algorithms can be beneficial to more efficiently predict mood based on HRV.

While HRV-based prediction of mood can contribute to early detection or objective assessment of mood disorders, caution is needed. Specific populations may have particular characteristics in mood. For example, perinatal women have prominent biological and psychosocial factors that affect mood, and are susceptible to mood disorders, including “maternity blues” and postpartum depression. However, HRV-based prediction for perinatal women has not been adequately addressed.

A common help-seeking barrier regarding postpartum depression was suggested to be women's inability to disclose their feelings (27). The previous study indicated that over 90% of women affected postpartum depression recognized there was something wrong, but only one-third believed they were suffering from postpartum depression, and over 80% had not reported their symptoms to any health professional (28). Cognitive behavioral interventions adapted for non-clinic settings during the perinatal period have effectively prevented postpartum depression (29). These suggest that perinatal women tend to fail to be aware of their emotions, and facilitating self-awareness of emotional conditions may be beneficial to prevent postpartum depression. During the perinatal period, most women experience sadness, anxiety, and frustration as representative negative emotions, which can be related to the physical and mental conditions resulting in mood disorders. It would be beneficial if emotions could be predicted based on HRV as an objective physiological marker. This would make it easier to record continuous alterations in daily life than using other biological indicators, such as body temperature, sweat, or blood pressure.

There have been several obstacles to predicting pregnant women's emotions based on HRV. Firstly, there has been no device that sufficiently records pregnant women's HRV in their daily life. Secondly, it has been controversial whether machine learning algorithms sufficiently predict emotions based on HRV, as previously described. To solve the first issue, we developed a system to record the HRV of pregnant women by attaching a small heart rate sensor to underwear for pregnant women. Using this device, we accumulated HRV of pregnant women along with simultaneous self-monitoring of four types of emotions: happiness, sadness, anxiety, and frustration. To solve the second issue, we conducted comprehensive evaluations of prediction accuracy of major currently available machine learning algorithms; the k-NN, SVM, LR, NB, SGD, DT, RF, GBT, XGBoost, and ANN, using the accumulated dataset. Thus, the purpose of this study was to (1) explore the algorithms that are most efficient in distinguishing and predicting the different emotional conditions of pregnant women and (2) evaluate the HRV features that are important in predicting different emotional conditions.

## Materials and Methods

**Figure 1:**
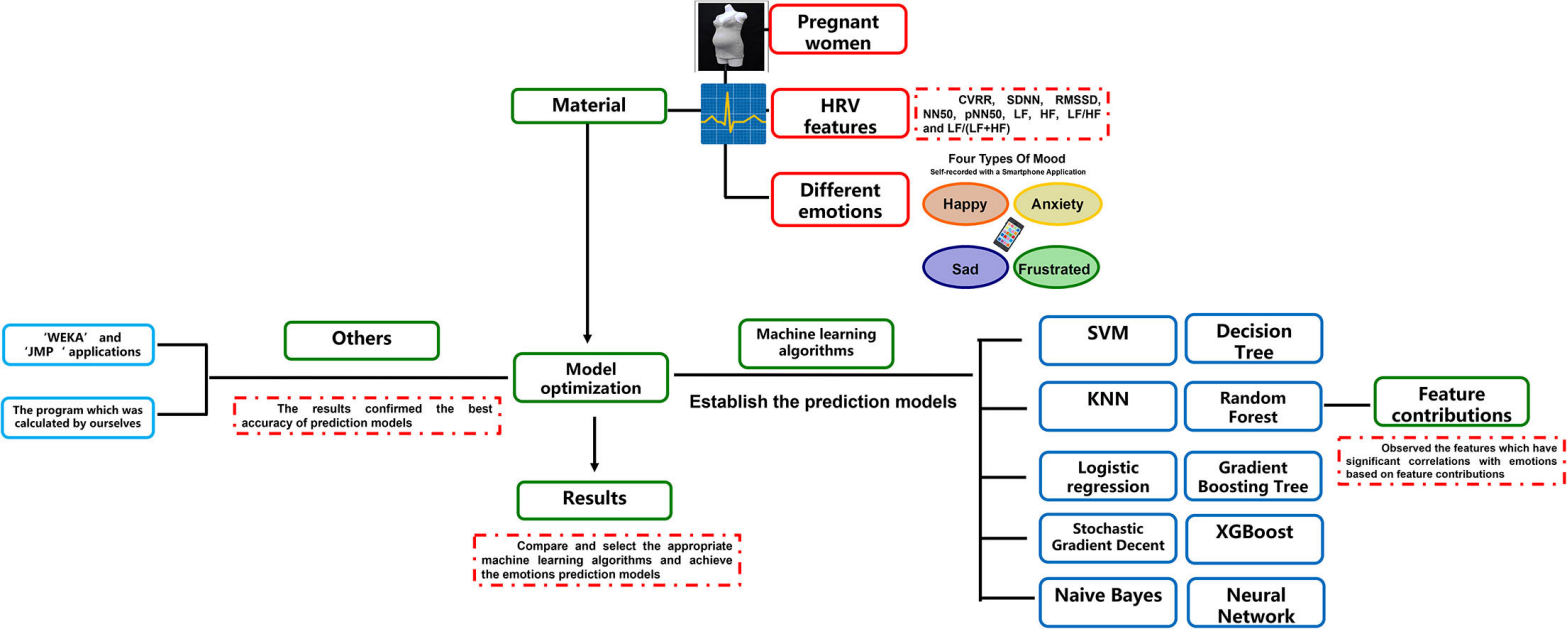
The design of this study. The design of this study was plotted. CVRR, coefficient of variation R-R interval; SDNN, standard deviation of all NN intervals; RMSSD, square root of the mean squared differences of successive NN intervals; NN50, number of interval differences of successive RR-intervals >50 ms; pNN50, the proportion derived by dividing NN50 by the total number of RR-intervals; LF, frequency domain features include low frequency; HF, high frequency; LF/HF, the ratio of low frequency to high frequency; SVM, support vector machine; k-NN, k-nearest neighbor; SGD, stochastic gradient descent; LR, logistic regression; DT, decision tree; NB, naïve Bayes; RF, random forest; GBT, gradient boosting trees; XGBoost, extreme gradient boosting; ANN, artificial neural network.

Figure 1 showed the design of this study included the materials (samples, HRV, and emotions) and methods (machine learning algorithms, feature contributions, and others). As shown in the figure, 10 algorithms were tested independently, and RF was indicated to produce the highest prediction accuracy.

### Data Collection

#### Participants

Participants were recruited from women who registered to a three-generation cohort of Tohoku Medical Megabank Organization during pregnancy. In the follow-up after delivery, a flier was distributed to notify women that they could participate in the current project when they became pregnant. From May 2018 to November 2019, 85 pregnant women, 25–44-years old, enrolled and completed the project. These women recorded their emotions for a collective total of 227.3 h (2.7 h per subject on average) during the period of observation from mid-pregnancy to 2 months after delivery. Emotional information was not available for 32 of the 85 perinatal women. Eleven participants recorded their emotions for <1 h, 25 for 1–3 h, five for 3–5 h, five for 5–8 h, and seven persons for over 8 h. Subjects were enrolled during week 23 of pregnancy (*n* = 4), week 24 (*n* = 4), week 25 (*n* = 14), week 26 (*n* = 17), week 27 (*n* = 15), week 28 (*n* = 12), week 29 (*n* = 6), week 30 (*n* = 2), week 31 (*n* = 3), and week 32 (*n* = 3). Forty-one of the 85 pregnant women gave birth to a baby girl and 43 to a baby boy, with no information for the remaining woman. Sixty-four deliveries were vaginal, and 20 delivered via Cesarean section. Two of the 85 women fed their babies with milk, 63 breast-fed, and 17 fed with a mixture of breast-feeding and milk. Thirty-four women were employed, 35 were unemployed, and 16 did not provide employment information. The study was approved by the Ethics Committee of Tohoku University (approval number: 2021-1-266). All women provided written informed consent.

#### Measures

##### Different Emotions

Participants reported their feelings via the application installed on their smartphones when they felt happy, anxious, sad, or frustrated by selecting their respective icons.

##### HRV

HRV data were obtained using a wearable heart rate monitor, MyBeat (UNIONTOOL, Tokyo), attached to the pregnant women's underwear (TOYOBO, OSAKA). The measured HRVs were the CVRR, SDNN, RMSSD, NN50, pNN50, LF, HF, and LF/HF. Supplementary Table 1 summarizes HRV descriptive information.

### Machine Learning Predictions of the Four Types of Emotions

The k-NN, SVM, LR, NB, SGD, DT, RF, GBT, XGBoost, and ANN machine learning algorithms (14, 18, 30–41) were applied to predict the four types of emotions based on HRV data. Summary explanations of the 10 algorithms are provided in Supplementary Text 2. The parameters of the models are listed in Supplementary Table 2. For the test set, we used the trained models to test and compare their prediction of emotions with real data (42, 43). The explanation of accuracy, precision, sensitivity, specificity, F1 score, and area under the receiver operating characteristic curve (AUC) is provided in Supplementary Text 3.

### Evaluations of Feature Contributions

RF was used to evaluate feature contributions to predicting four types of emotions. Feature analysis evaluated all features and observed the important features that had significant correlations with the different types of emotions based on feature contributions. Thus, as used in the previous studies, RF was used as a classifier (44–46) and a method to evaluate feature contributions (44, 47, 48).

### Validation of the Analyses

#### Alternative Applications for Machine Learning Predictions

Waikato Environment for Knowledge Analysis, University of Waikato, New Zealand (WEKA) and JMP statistical software (SAS Institute, Cary, NC, USA) were used to analyze the same dataset and prediction models.

#### Alternative Calculations of HRV Indicators

We primarily used the HRV indicators calculated using the program installed in the MyBeat device. The source codes of the algorithms used to calculate the HRV indicators in the device are proprietary. To validate the HRV indicators provided by the device, we calculated HRV indicators in python using open-source codes. The multiple formulae used to calculate time domain features included CVRR, SDNN, RMSSD, NN50, and pNN50, and frequency domain features that included LF and HF. The formula used to calculate the remaining HRV indicators is summarized in Supplementary Text 1 (49–54). The HRV indicators given by MyBeat were compared with those calculated using python to ensure consistency between the two.

#### Cross-Validations of Models for Hyper-Parameter Search

To validate the aforementioned machine learning algorithms to construct prediction models of the different emotions, samples were randomly split into two groups to generate the training dataset and the test dataset (55–57) and subjected to cross-validation. To select the most appropriate cross-validation method, k-fold cross-validation (KCV) (58–60) (test size = 0, *k* = 5), GridSearch Cross-validation (GridSearchCV) (61–63), and RandomizedSearch Cross-validation (RandomizedSearchCV) (64–66) were tested in a preliminary study. RandomizedSearchCV provided the highest accuracy with the fastest calculation time. The optimal parameters are listed in Supplementary Table 2.

## Results

### Profiles of Collected Data

Among the 85 perinatal women, 32 did not input any emotion information on the smartphone application. The remaining 53 perinatal women recorded one of the four types of emotions when they felt that emotion during the 1 week in the observation period when their heart rates were monitored. On average, during the week, each subject recorded “happy” for 1.92 h, “frustrated” for 1.59 h, “anxious” for 0.47 h, and “sad” for 0.31 h.

### Machine Learning Predictions of the Four Types of Emotions

Among the 10 machine learning algorithms applied to predict four types of emotions based on HRV indicators. RF showed the highest AUC of 0.70, followed by GBT (0.69), ANN (0.68), XGBoost (0.66), SVM (0.65), LR (0.65), DT (0.65), SGD (0.64), k-NN (0.61), and NB (0.52). The accuracy, precision, sensitivity, F1 score, and AUC of the 10 machine learning algorithms are summarized in Table 1. ROC curve of Random Forest is shown in Supplementary Figure 1, and the accuracy of training and test dataset with Random Forest is shown in Supplementary Figure 2.

**Table 1:** Model evaluation indices of the 10 machine learning prediction of the four selected emotions.

**Items**	**SVM**	**k-NN**	**SGD**	**LR**	**DT**	**NB**	**RF**	**GBT**	**XGBoost**	**ANN**
Accuracy	0.72	0.73	0.72	0.73	0.73	0.61	0.74	0.73	0.72	0.74
Precision	0.66	0.68	0.66	0.67	0.67	0.67	0.69	0.67	0.66	0.68
Sensitivity	0.72	0.73	0.72	0.73	0.73	0.61	0.74	0.73	0.72	0.74
F1 score	0.66	0.68	0.66	0.66	0.68	0.63	0.69	0.68	0.68	0.68
AUC	0.65	0.61	0.64	0.65	0.65	0.52	0.70	0.69	0.66	0.68

*The model evaluation indices of the 10 machine learning predictions of the four emotions used the test dataset (5-fold cross-validation) independent of the training dataset*.

*SVM, support vector machine; k-NN, k-nearest neighbor; SGD, stochastic gradient descent; LR, logistic regression; DT, decision tree; NB, naïve Bayes; RF, random forest; GBT, gradient boosting trees; XGBoost, extreme gradient boosting; ANN, artificial neural network; AUC, area under the curve*.

### Runtime Efficiency of Each Machine Learning Algorithm

The runtime efficiency was measured for each prediction model. All the models were completed within 3–12 s. NB took 3 s, RF and LR took 4 s, k-NN took 6 s, SVM took 7 s, DT took 8 s, SGD took 9 s, GBT, XGBoost, and ANN took 12 s.

### Evaluations of Each Feature

**Figure 2:**
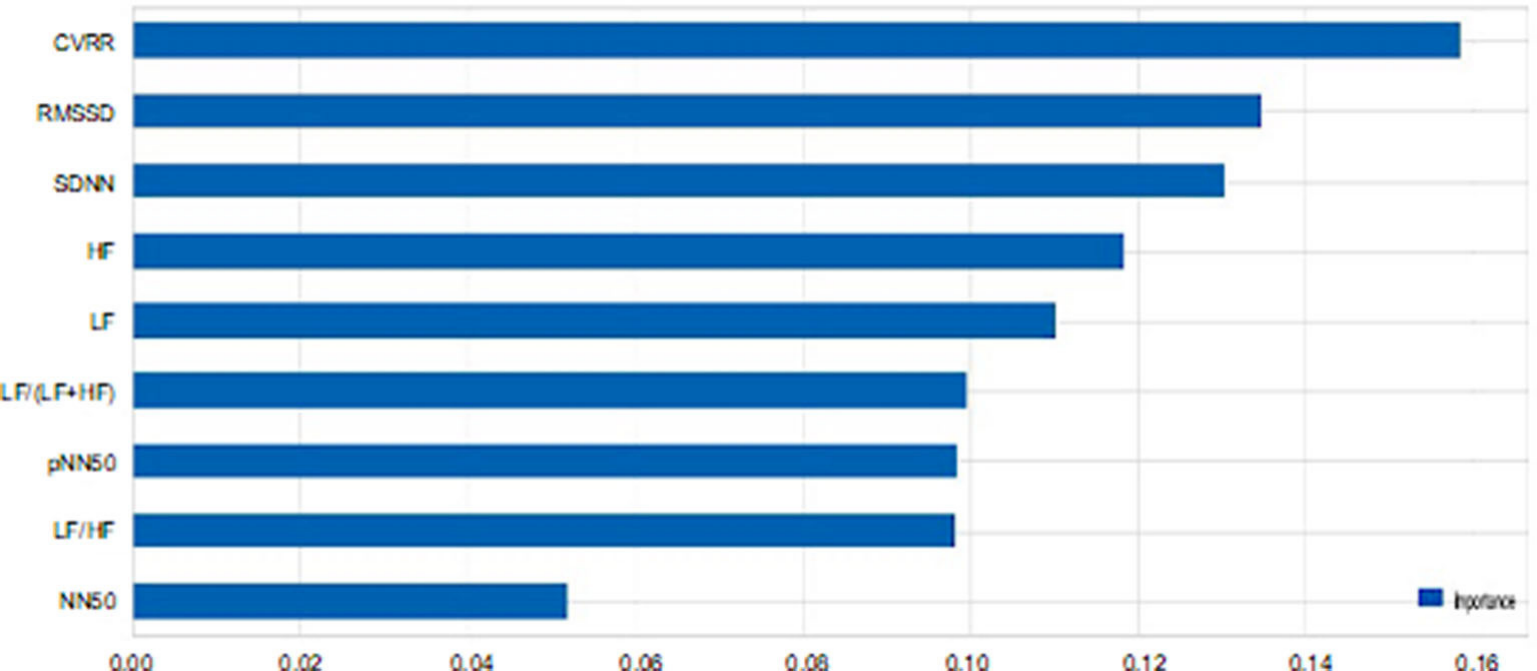
Importance of each heart rate variability indicator. The importance scores of each feature in the prediction of emotions based on the nine heart rate variability indicators using random forest are plotted. CVRR, coefficient of variation R-R interval; SDNN, standard deviation of all NN intervals; RMSSD, square root of the mean squared differences of successive NN intervals; NN50, number of interval differences of successive RR-intervals >50 ms; pNN50, the proportion derived by dividing NN50 by the total number of RR-intervals; LF, frequency domain features include low frequency; HF, high frequency; LF/HF, the ratio of low frequency to high frequency.

**Figure 3:**
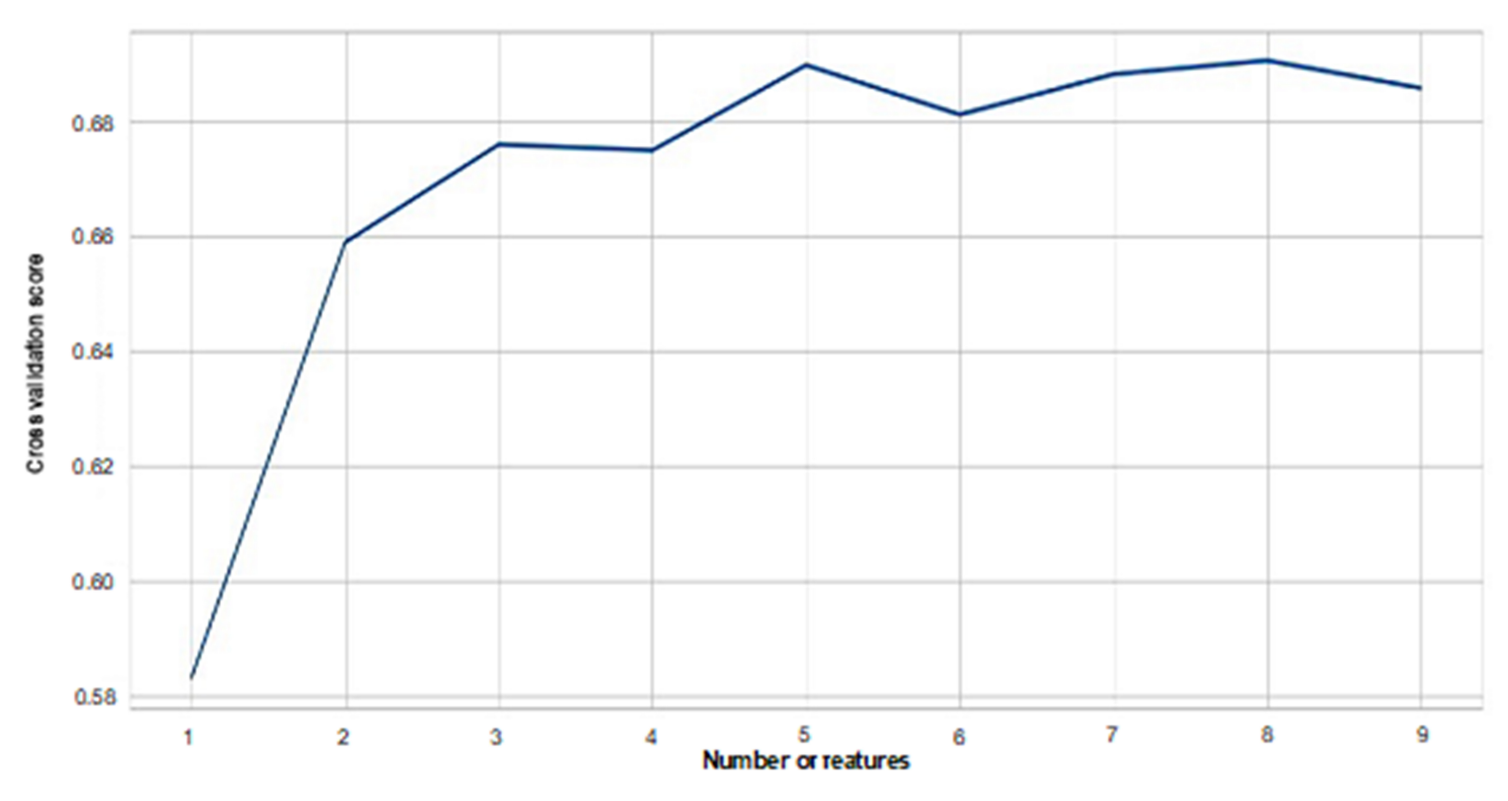
Numbers of features and cross-validation scores of random forest-based prediction of emotions. Cross-validation scores for each number of features used in the prediction of emotions are plotted. As more features are included in the prediction, cross-validation scores increase. A plateau is reached when five features are included.

Feature evaluation of the nine HRV indicators using RF revealed that CVRR showed the highest important score in the prediction of emotions, followed by RMSSD, SDNN, HF, LF/(LF+HF), pNN50, LF/HF, and NN50 showed the lowest contribution to the prediction (Figure 2). Cross-validation scores were plotted with the number of features used to predict emotions. When features with higher contributions were added to the predictions one by one, the cross-validation scores increased as more features were included in the prediction up to five features. After more than five features were included, the prediction accuracy reached the plateau, as shown in Figure 3.

### Validation of Analyses

WEKA and JMP analyses of the same dataset produced the same results regarding the AUC of the predictions using the 10 algorithms. In addition, predictions of emotions using the source codes of algorithms to calculate HRV indicators provided the same results of predictions using the HRV indicators produced by the program installed in the MyBeat device. The results of other applications are provided in Supplementary Tables 3, 4 and Supplementary Figure 3.

To validate the ability of the aforementioned machine learning algorithms in building prediction models of the different emotions, KCV, GridSearchCV, and RandomizedSearchCV were tested in a preliminary study. As explained in section Cross-Validations of Models for Hyper-Parameter Search, RandomizedSearchCV was selected. The optimal parameters are listed in Table 2.

**Table 2:** Optimal parameters.

**Machine learning algorithm**	**Important parameters**
SVM	*C* = 1.0, kernel = “rbf,” penalty = “l2”
KNN	n_neighbors = 8, *p* = 2
LR	penalty = “l2,” “class_weight”: None
NB	var_smoothing = 1e-09
SGD	penalty = “l2,” alpha = 0.001
GBT	n_estimators = 100, criterion = “friedman_mse”
XGBoost	max_depth = 13, gamma = 2, objective = “multi:softmax”
DT	max_depth = 10, criterion = “gini,” splitter = “random,”
RF	n_estimators = 200, criterion = “gini,” max_features = 5
ANN	hidden_layer_sizes = (100,), alpha = 0.001, max_iter = 200

*SVM, support vector machine; k-NN, k-nearest neighbor; SGD, stochastic gradient descent; LR, logistic regression; DT, decision tree; NB, naïve Bayes; RF, random forest; GBT, gradient boosting trees; XGBoost, extreme gradient boosting; ANN, artificial neural network; AUC, area under the curve*.

## Discussion

Unlike the previous studies, which applied a limited number of machine learning algorithms to predict emotions based on HRV indicators, the current study firstly conducted comprehensive evaluations of widely used algorithms. Among the python-based predictions of the happy, anxious, sad, and frustrated emotions using 10 machine learning algorithms, RF provided the highest AUC, followed closely by GBT. Their AUC values were higher than those of the remaining eight algorithms (SVM, k-NN, NB, ANN, DT, XGBoost, SGD, and LR), as summarized in Supplementary Table 5 along with their characteristics.

There are some discrepancies between the results of previous studies and the present study. Table 3 summarizes the validation designs of the previous studies and the algorithms that showed the best prediction accuracies. Previous studies reported the superiority of SVM (5, 12–15), k-NN (18, 19), LR (16, 17), and ANN (26). There are several possible reasons for these discrepancies. First, SVM has parameters that include C and gamma. Setting a higher value of gamma can cause overfitting problems (i.e., high accuracy in the training dataset and low accuracy in the test dataset). While datasets independent from training datasets should be used as test datasets to validate the accuracy of algorithms, some previous studies did not detail whether the validation was properly performed using independent datasets. Second, SVM is a proper method to recognize two types of emotions, positive emotions and negative emotions (12). The present study intended to differentiate four selected types of emotions. Third, the sample sizes of some of the prior studies were relatively small, which resulted in lower reliability. Fourth, we tested multiple methods and selected RandomizedSearchCV for cross-validations, which provided the best prediction accuracies for each algorithm. Whether the previous studies used proper cross-validations for their algorithms is unclear, because some of the studies did not describe how to conduct cross-validations.

**Table 3:** Previous machine learning studies to predict emotions based on heart rate variabilities.

**First author (reference citation)**	**Year of publication**	**Training dataset (n)**	**Test dataset (n)**	**Method of cross-validation**	**Total sample size (n)**
Rakshit et al. (5)	2016	33	N.A.	Leave-one-out cross-validation	33
Cheng et al. (12)	2017	N.A	N.A	N.A	N.A
Jang et al. (13)	2012	N.A	N.A	N.A	200
Guo et al. (14)	2016	N.A	N.A	N.A.	25
Domínguez-Jiménez et al. (15)	2020	80% of total number of subjects	20% of total number of subjects	N.A.	37
Chueh et al. (16)	2012	10	2	Leave-one-out cross-validation	12
Shu et al. (17)	2020	25	N.A.	Leave-one-out cross-validation	25
Zheng et al. (18)	2012	20	10-fold cross-validation	10-fold cross-validation	20
Ferdinando et al. (19)	2016	80% of total number of subjects	20% of total number of subjects	N.A	512
Jang et al. (20)	2014	70% of total number of subjects	30% of total number of subjects	N.A.	300
Subramanian et al. (21)	2016	N.A	N.A.	A leave-one-out cross-validation	58
Nikolova et al. (22)	2019	N.A	N.A	N.A	25
Colomer Granero et al. (23)	2016	47	N.A.	10-fold cross-validation	47
Ayata et al. (24)	2018	32	N.A.	10-fold cross-validation	32
Su et al. (25)	2020	369,289 records	41,033 records	N.A	25
Lee et al. (26)	2005	N.A	N.A	N.A	6
**First author (reference citation)**	**SVM**	**k-NN**	**LR**	**SGD**	**NB**	**DT**	**RF**	**GBT**	**XGBoost**	**ANN**
Rakshit et al. (5)	*	-	-	-	-	-	-	-	-	-
Cheng et al. (12)	*	o	-	-	-	o	o	o	-	-
Jang et al. (13)	*	-	-	-	-	o	-	-	-	-
Guo et al. (14)	*	-	-	-	-	-	-	-	-	-
Domínguez-Jiménez et al. (15)	*	o	o	-	o	o	o	-	o	-
Chueh et al. (16)	o	o	*	-	o	o	-	-	-	-
Shu et al. (17)	-	-	*	-	-	-	-	-	-	o
Zheng et al. (18)	-	*	-	-	-	-	-	-	-	-
Ferdinando et al. (19)	-	*	-	-	-	-	-	-	-	-
Jang et al. (20)	o	-	-	-	*	o	-	-	-	-
Subramanian et al. (21)	o	-	-	-	*	-	-	-	-	-
Nikolova et al. (22)	o	-	-	-	*	-	-	-	-	o
Colomer Granero et al. (23)	o	-	o	-	o	-	*	-	-	o
Ayata et al. (24)	o	o	-	-	-	o	*	-	-	-
Su et al. (25)	-	o	-	-	-	o	*	*	-	-
Lee et al. (26)	-	-	-	-	-	-	-	-	-	*

o, machine learning algorithm tested in the study; -, machine learning algorithm not tested in the study; *machine learning algorithm with the highest prediction accuracy in the study. SVM, support vector machine; k-NN, k-nearest neighbor; SGD, stochastic gradient descent; LR, logistic regression; DT, decision tree; NB, naïve Bayes; RF, random forest; GBT, gradient boosting trees; XGBoost, extreme gradient boosting; ANN, artificial neural network.

It is noteworthy that SVM, k-NN, LR, ANN, and NB belong to the classifier type of machine learning algorithms, whereas RF and GBT belong to the ensemble type of machine learning algorithms. Whether classifier (7) or ensemble is suitable for physiological signal-based emotion recognition is contentious. While most of the previous studies claimed that their proposed method was superior compared with other competitive methods, drawbacks of some studies included the aforementioned small sample sizes and lack of proper cross-validations. Ensemble algorithms, such as RF and GBT, analyze high-dimensional data and solve a variety of problems to achieve high accuracy (24). This contrasts with simple classifiers, such as SVM and k-NN, which are suitable for small sample sizes.

In the present study, the RF and GBT ensemble learning algorithms displayed the best AUCs predicting the selected emotions. This is probably because these algorithms can solve and deal with overfitting problems to achieve the best balance between generalization and regularization (25, 67–70).

The strengths of other algorithms are listed in Supplementary Table 5. These algorithms may have shortcomings in the prediction of four types of emotions based on HRV in the present study. For example, LR and SVM are appropriate methods to predict binary information but are not suitable to predict more than two types of information, such as four types of emotions considered in our research. In addition, LR, SVM, k-NN, and DT have difficulty analyzing large amounts of data, especially because they include many characteristic numbers and unbalanced data distribution. NB and DT are based on the assumption of the independence of sample attributes and so are not suitable for data in which sample attributes are related to one another, such as our data. In addition, LR, NB, and SGD generally have low accuracy and a high error rate of classification decisions (71). ANN generally has many parameters, such as the network topology, weights, and thresholds, which are difficult to regulate. In addition, the output results are difficult to explain, which affects the credibility and acceptability of the results.

The run time of RF was relatively shorter than other algorithms. Considering the high AUC and short runtime may suggest the usability of RF in the simultaneous prediction of emotions in daily activities.

Concerning cross-validation methods, GridSearchCV can ensure that the accuracy of the parameters is within the specified parameter range by traversing all possible combinations of parameters. This is very time-consuming in the case of large datasets and multiple parameters. Randomized SearchCV has supplanted GridSearchCV by random sampling in the parameter space. For parameters with continuous variables, RandomizedSearchCV samples them as a distribution, whose search ability depends on the n_iter set parameter. Bergstra et al. (72) proved empirically and theoretically that RandomizedSearchCV is more effective than grid search in hyper-parameter optimization.

RF was used to evaluate the important features to predict the four types of emotions. Feature analysis based on RF indicated that CVRR, RMSSD, SDNN, HF, and LF, among the nine HRV indicators, were important features to predict emotions. HRV has been considered as a marker of emotional response as per many theories, among which the polyvagal theory and the model of neurovisceral integration are the main supporting theories (73). The nine indicators of HRV used in this study were previously analyzed in the context of numerous psychological and physical health concerns. Through these studies, these indicators were proposed as biomarkers of capacity for the self-regulation of physiological, emotional, and cognitive responses and for effective adaptation to environmental stress and demands. Wang et al. found that SDNN, CVRR, and LF of subjects who had negative emotions (fear, stress, and anxiety) were higher than those who had positive emotions. The study also showed that HF of those with negative emotions was lower than that of those who had positive emotions (74). Simplicio et al. found that decreased HF was correlated with a loss of flexibility in the parasympathetic cardiovascular tone and emotion regulation. RMSSD reflects parasympathetic nerve activity (75). Godfrey et al. (3) reported that RMSSD decreased under mental stress. Zhu et al. reported that SDNN, RMSSD, LF, and HF are related to emotions (76). Thus, all of these five HRV indicators have been suggested to reflect emotions (77–80), which was confirmed in the present study.

The present data indicated that other features (NN50, pNN50, and LF/HF) were not as important as the five HRV indicators to predict the four types of emotions. The mechanical interpretation of the findings is difficult. Firstly, RR-intervals are affected by multiple control mechanisms, including autonomic modulation at the sinoatrial node, dynamic regulation of the vasculature, and endocrine/paracrine, endothelial, and mechanical factors. Additionally, complex control mechanisms, including baroreflex and respiratory sinus arrhythmia, can also drive changes in these parameters. While various studies have revealed CVRR, SDNN, and RMSSD as indicators responsive to different emotions, NN50 has been studied or proven not to be correlated with autonomic nervous function (6, 75, 81). In addition, while many studies suggest that LF and HF reflect other mechanisms that exert regulatory control over the cardiac cycle, such as baroreflex activity in response to vasomotor tone (82, 83). The findings that LF/HF may be an indicator to reflect the balance of the sympathetic and parasympathetic nerves (84) has become controversial. For example, a recent analysis of this metric cast doubt on its interpretation (85).

We simultaneously tested different applications (JMP and WEKA) to analyze the data with algorithms, because the different applications provided slightly different results owing to variability in parameter regulations. In addition, we used Welch's method in python to extract the HRV based on RR-intervals. These alternative analyses provided the consistent finding that RF was the most appropriate method among the tested algorithms, which confirmed the best prediction accuracy of RF.

## Limitations

This study has several limitations. First, the sample size was still relatively small (*n* = 85), although it was more extensive than previous studies. In addition, the observations of sadness and anxiety were less frequent than those of the other two emotions. Further investigation using more data will be needed to verify the accuracy of the model. Second, there was a potential selection bias. For example, perinatal women who had a more vital interest in maintaining their health conditions might have tended to enroll in the study. Third, there might be variability in self-recording of emotions among the participants regarding sensitivity to the alterations in their mood or diligence to record their emotions. Finally, the traditional methods for machine learning and optimization were used in the current study. Deep learning algorithms were not applicable due to the small number of observations of sadness and anxiety. After collecting more data, advanced methods such as deep learning algorithms (86–90) and Bayesian optimization (86) would be applicable in the future to optimize prediction models and the parameters.

## Conclusions

Comprehensive analyses of 10 machine learning algorithms indicated that RF and GBT provided the highest prediction accuracy and suggested the usability of the algorithms to predict emotions based on autonomic nervous system indicators of pregnant women. The results also implicated SDNN, RMSSD, CVRR, LF, and HF as important parameters for the predictions.

## Data Availability Statement

The raw data supporting the conclusions of this article will be made available by the authors, without undue reservation.

## Ethics Statement

The studies involving human participants were reviewed and approved by the Ethics Committee of Tohoku University. The patients/participants provided their written informed consent to participate in this study.

## Author Contributions

CO, NW, TS, TaN, HU, KM, MI, TO, FN, NF, ShK, MY, NY, and HT contributed to the acquisition of data. CO, ToN, TT, SO, and GT contributed to the data management. XL, CO, ZY, YT, KI, NS, NH, and HT contributed to the data analysis. XL, CO, KI, NS, NK, SaK, YK, JS, TH, MS, ShK, NH, and HT contributed to the interpretation of the data. XL and HT were involved in drafting the manuscript. HT, KI, NS, NK, and NH critically revised the manuscript for important scientific content. XL, CO, NW, FN, ShK, and HT made substantial contributions to the conception and design of the study. All authors listed have made a substantial, direct, and intellectual contribution to the work and approved it for publication.

## Funding

This research was supported by a grant from the Strategic Research Program for Brain Sciences from the Japan Agency for Medical Research and Development (AMED) under (Grant Number JP20dm0107099), the Tohoku Medical Megabank Project from the Ministry of Education, Culture, Sports, Science and Technology (MEXT) of Japan and AMED under (Grant Numbers JP20km0105001 and JP20km0105002), and Tohoku University Advanced Research Center for Innovations in Next-Generation Medicine. We are grateful to the project participants for supporting this study.

## Conflict of Interest

The authors declare that the research was conducted in the absence of any commercial or financial relationships that could be construed as a potential conflict of interest.

## Publisher's Note

All claims expressed in this article are solely those of the authors and do not necessarily represent those of their affiliated organizations, or those of the publisher, the editors and the reviewers. Any product that may be evaluated in this article, or claim that may be made by its manufacturer, is not guaranteed or endorsed by the publisher.

## References

[1] Acharya UR Joseph KP Kannathal N Lim CM Suri JS. Heart rate variability: a review. Med Biol Eng Comput. (2006) 44:1031–51. 10.1007/s11517-006-0119-0 17111118

[2] Gouizi K Maaoui C Reguig FB. Negative emotion detection using EMG signal. in 2014 International Conference on Control, Decision and Information Technologies. Nancy: IEEE (2014). pp. 690–5.

[3] Godfrey KM Juarascio A Manasse S Minassian A Risbrough V Afari N. Heart rate variability and emotion regulation among individuals with obesity and loss of control eating. Physiol Behav. (2019) 199:73–8. 10.1016/j.physbeh.2018.11.009 30414883 PMC6492031

[4] Xiu L Zhou R Jiang Y. Working memory training improves emotion regulation ability: evidence from HRV. Physiol Behav. (2016) 155:25–9. 10.1016/j.physbeh.2015.12.004 26679738

[5] Rakshit R Reddy VR Deshpande P. Emotion detection and recognition using HRV features derived from photoplethysmogram signals. in Proceedings of the 2nd Workshop on Emotion Representations and Modelling for Companion Systems. Tokyo (2016). pp 1–6.

[6] Goldstein DS Bentho O Park MY Sharabi Y. Low-frequency power of heart rate variability is not a measure of cardiac sympathetic tone but may be a measure of modulation of cardiac autonomic outflows by baroreflexes. Exp Physiol. (2011) 96:1255–61. 10.1113/expphysiol.2010.056259 21890520 PMC3224799

[7] Li W Zhang Z Song A. Physiological-signal-based emotion recognition: an odyssey from methodology to philosophy. Measurement. (2021) 172:108747. 10.1016/j.measurement.2020.108747

[8] Wen W Liu G Cheng N Wei J Shangguan P Huang W. Emotion recognition based on multi-variant correlation of physiological signals. IEEE Trans Affect Comput. (2014) 5:126–40. 10.1109/TAFFC.2014.232761727295638

[9] Ayata D Yaslan Y Kamasak ME. Emotion recognition from multimodal physiological signals for emotion aware healthcare systems. J Med Biol Eng. (2020) 40:1–9. 10.1007/s40846-019-00505-7

[10] Zhang Y Cheng C Chen T. Multi-channel physiological signal emotion recognition based on relieff feature selection. in 2019 IEEE 25th International Conference on Parallel and Distributed Systems (ICPADS). Tianjin: IEEE (2019). pp 725–30.

[11] Shin D Shin D Shin D. Development of emotion recognition interface using complex EEG/ECG bio-signal for interactive contents. Multimed Tools Appl. (2017) 76:11449–70. 10.1007/s11042-016-4203-7

[12] Cheng Z Shu L Xie J Chen CP. A novel ECG-based real-time detection method of negative emotions in wearable applications. in 2017 International Conference on Security, Pattern Analysis, and Cybernetics (SPAC). Shenzhen: IEEE (2017). pp. 296–301.

[13] Jang E-H Rak B Kim S-H Sohn J-H. Emotion classification by machine learning algorithm using physiological signals. Proc Comput Sci Inf Technol Singapore. (2012) 25:1–5. 10.1109/ICNSC.2012.620493127295638

[14] Guo H-W Huang Y-S Lin C-H Chien J-C Haraikawa K Shieh J-S. Heart rate variability signal features for emotion recognition by using principal component analysis and support vectors machine. in 2016 IEEE 16th International Conference on Bioinformatics and Bioengineering (BIBE). Taichung: IEEE (2012). pp. 274–7.

[15] Dominguez-Jimenez JA Campo-Landines KC Martínez-Santos JC Delahoz EJ Contreras-Ortiz SH. A machine learning model for emotion recognition from physiological signals. Biomed Signal Process Control. (2020) 55:101646. 10.1016/j.bspc.2019.101646

[16] Chueh T-H Chen T-B Lu HH-S Ju S-S Tao T-H Shaw J-H. Statistical prediction of emotional states by physiological signals with manova and machine learning. Int J Pattern Recogn Artif Intell. (2012) 26:1250008. 10.1142/S0218001412500085

[17] Shu L Yu Y Chen W Hua H Li Q Jin J Xu X. Wearable Emotion Recognition Using Heart Rate Data from a Smart Bracelet. Sensors. (2020) 20:718. 10.3390/s20030718 32012920 PMC7038485

[18] Zheng BS Murugappan M Yaacob S. Human emotional stress assessment through Heart Rate Detection in a customized protocol experiment. 2012 IEEE Symposium on Industrial Electronics and Applications. Bandung: IEEE (2012). pp. 293–8.

[19] Ferdinando H Seppänen T Alasaarela E. Comparing features from ECG pattern and HRV analysis for emotion recognition system. 2016 IEEE Conference on Computational Intelligence in Bioinformatics and Computational Biology (CIBCB). Chiang Mai: IEEE (2012). pp. 1–6.

[20] Jang E-H Park B-J Kim S-H Chung M-A Park M-S Sohn J-H. Emotion classification based on bio-signals emotion recognition using machine learning algorithms. in 2014 International Conference on Information Science, Electronics and Electrical Engineering. Sapporo City: IEEE (2014). pp. 1373–6.

[21] Subramanian R Wache J Abadi MK Vieriu RL Winkler S Sebe N. Emotion and personality recognition using commercial sensors. IEEE Trans Affect Comput. (2016) 9:147–60. 10.1109/TAFFC.2016.262525027295638

[22] Nikolova D Mihaylova P Manolova A Georgieva P. ECG-Based Human Emotion Recognition Across Multiple Subjects. in International Conference on Future Access Enablers of Ubiquitous and Intelligent Infrastructures. Cham: Springer (2019). pp. 25–36.

[23] Colomer Granero A Fuentes-Hurtado F Naranjo Ornedo V Guixeres Provinciale J Ausín JM Alcañiz Raya M. comparison of physiological signal analysis techniques and classifiers for automatic emotional evaluation of audiovisual contents. Front Comput Neurosci. (2016) 10:74. 10.3389/fncom.2016.00074 27471462 PMC4945646

[24] Ayata D Yaslan Y Kamasak ME. Emotion based music recommendation system using wearable physiological sensors. IEEE Trans Consum Electron. (2018) 64:196–203. 10.1109/TCE.2018.284473627295638

[25] Su C. Heart Rate Variability Feature Selection using Random Forest for Mental Stress Quantification. Montreal, QC: Concordia University (2020).

[26] Lee C Yoo S Park Y Kim N Jeong K Lee B. Using neural network to recognize human emotions from heart rate variability and skin resistance. in 2005 IEEE Engineering in Medicine and Biology 27th Annual Conference. Shanghai: IEEE (2005). pp. 5523–5. 10.1109/IEMBS.2005.1615734 17281504

[27] Dennis Cindy-Lee Leinic Chung-Lee. Postpartum depression help-seeking barriers and maternal treatment preferences: a qualitative systematic review. Birth. (2006) 33:323–31. 10.1111/j.1523-536X.2006.00130.x 17150072

[28] Whitton Anna Rachel Warner and Louis Appleby. The pathway to care in post-natal depression: women's attitudes to post-natal depression and its treatment. Br J Gen Pract. (1996)46: 427–8. 8776916 PMC1239697

[29] Sockol LE. A systematic review of the efficacy of cognitive behavioral therapy for treating and preventing perinatal depression. J Affect Disord. (2015) 177:7–21. 10.1016/j.jad.2015.01.052 25743368

[30] Zhang W Liu H Silenzio VMB Qiu P Gong W. Machine learning models for the prediction of postpartum depression: application and comparison based on a cohort study. JMIR Med Inf. (2020) 8:15516. 10.2196/15516 32352387 PMC7226048

[31] Urtnasan E Park J-U Lee S Lee K-J. Optimal classifier for detection of obstructive sleep apnea using a heartbeat signal. Int J Fuzzy Logic Intell Syst. (2017) 17:76–81. 10.5391/IJFIS.2017.17.2.76

[32] Breiman L. Random forests. Mach Learn. (2001) 45:5–32. 10.1023/A:1010933404324

[33] Xiao M Yan H Song J Yang Y Yang X. Sleep stages classification based on heart rate variability and random forest. Biomed Signal Process Control. (2013) 8:624–33. 10.1016/j.bspc.2013.06.001

[34] Lee HG Noh KY Ryu KH. Mining biosignal data: coronary artery disease diagnosis using linear and nonlinear features of HRV. in Pacific-Asia Conference on Knowledge Discovery and Data Mining Springer. Gold Coast QLD (2007). pp. 218–28.

[35] Natarajan S Prabhakar A Ramanan N Bagilone A Siek K Connelly K. Boosting for postpartum depression prediction. in 2017 IEEE/ACM International Conference on Connected Health: Applications, Systems and Engineering Technologies (CHASE). Philadelphia, PA: IEEE (2017). pp. 232–40.

[36] Plewa L Student C P S L O. iStress: Stress Classification From Heart Rate Variability. Cal Poly: CSC 520 96 Spring (2015).

[37] Bottou L Bousquet O. The Tradeoffs of Large Scale Learning, Advances in Neural Information Processing Systems. Cambridge, MA: MIT Press (2008).

[38] Wang S Pathak J Zhang Y. Using electronic health records and machine learning to predict postpartum depression. Stud Health Technol Inf. (2019) 264:888–92. 10.3233/SHTI190351 31438052

[39] Kelwade J Salankar S. Prediction of cardiac arrhythmia using artificial neural network. Int J Comput Appl. (2015) 115:30–5. 10.5120/20270-267914684265

[40] Yoo SK Lee CK Park YJ Kim NH Lee BC Jeong KS. Neural network based emotion estimation using heart rate variability and skin resistance. in International Conference on Natural Computation. Berlin: Springer (2005). pp. 818–24.

[41] Joo S Choi K-J Huh S-J. Prediction of spontaneous ventricular tachyarrhythmia by an artificial neural network using parameters gleaned from short-term heart rate variability. Expert Syst Appl. (2012) 39:3862–6. 10.1016/j.eswa.2011.09.097

[42] Sokolova M Lapalme G A. systematic analysis of performance measures for classification tasks. Inf Process Manag. (2009) 45:427–37. 10.1016/j.ipm.2009.03.00232003740

[43] Bowes D Hall T and Gray D. Comparing the performance of fault prediction models which report multiple performance measures: recomputing the confusion matrix. in Proceedings of the 8th International Conference on Predictive Models in Software Engineering. Lund (2012). pp. 109–18.

[44] Palczewska A Palczewski J Robinson RM Neagu D. Interpreting random forest classification models using a feature contribution method. in Integration of Reusable Systems. San Francisco, CA: Springer (2014). pp. 193–218.

[45] Mursalin M Zhang Y Chen Y Chawla NV. Automated epileptic seizure detection using improved correlation-based feature selection with random forest classifier. Neurocomputing. (2017) 241:204–14. 10.1016/j.neucom.2017.02.053

[46] Paul D Su R Romain M Sébastien V Pierre V Isabelle G. Feature selection for outcome prediction in oesophageal cancer using genetic algorithm and random forest classifier. Computer Med Imaging Graph. (2017) 60:42–9. 10.1016/j.compmedimag.2016.12.002 28087102

[47] Palczewska A Palczewski J Robinson RM Neagu D. Interpreting random forest models using a feature contribution method. in 2013 IEEE 14th International Conference on Information Reuse & Integration (IRI). San Francisco, CA: IEEE (2013). pp. 112–9.

[48] Whitmore LS George A Hudson CM. Explicating feature contribution using random forest proximity distances. In: arXiv preprint arXiv:180706572. IJCAI/ECAI 2018 Workshop on Explainable Artificial Intelligence (XAI) (2018).

[49] Wang H-M Huang S-C. SDNN/RMSSD as a surrogate for LF/HF: a revised investigation. Modell Simul Eng. (2012) 2012:16. 10.1155/2012/9319432012

[50] Doret M Spilka J Chudáček V Gonçalves P Abry P. Fractal analysis and hurst parameter for intrapartum fetal heart rate variability analysis: a versatile alternative to frequency bands and LF/HF ratio. PLoS ONE. (2015)10:136661. 10.1371/journal.pone.0136661 26322889 PMC4556442

[51] Schaffer T Hensel B Weigand C Schüttler J Jeleazcov C. Evaluation of techniques for estimating the power spectral density of RR-intervals under paced respiration conditions. J Clin Monitor Comput. (2014) 28:481–6. 10.1007/s10877-013-9447-4 23508826

[52] Posada-Quintero HF Florian JP Orjuela-Cañón AD Aljama-Corrales T Charleston-Villalobos S Chon KH. Power spectral density analysis of electrodermal activity for sympathetic function assessment. Ann Biomed Eng. (2016) 44:3124–35. 10.1007/s10439-016-1606-6 27059225

[53] Verma A Cabrera S Mayorga A Nazeran H. A robust algorithm for derivation of heart rate variability spectra from ECG and PPG signals. in 29th Southern Biomedical Engineering Conference. Miami, FL: IEEE (2013). pp. 35–6.

[54] Glos M Romberg D Fietze I Rottig J Knobe M Witt C. Analysis of heart rate and blood pressure variability during nasal continuous positive airway pressure therapy in patients with obstructive sleep apnea. in Computers in Cardiology. Hannover: IEEE (1999). pp. 603–5.

[55] Pourghasemi HR Pradhan B Gokceoglu C. Application of fuzzy logic and analytical hierarchy process (AHP) to landslide susceptibility mapping at Haraz watershed, Iran. Nat Hazards. (2012) 63:965–96. 10.1007/s11069-012-0217-2

[56] Pham BT Bui D Prakash I Dholakia M. Evaluation of predictive ability of support vector machines and naive Bayes trees methods for spatial prediction of landslides in Uttarakhand state (India) using GIS. J Geomat. (2016) 10:71–9. Available online at: https://isgindia.org/JOG/abstracts/APR-2016/pap012.pdf

[57] Huang F Zhang J Zhou C Wang Y Huang J Zhu L. deep learning algorithm using a fully connected sparse autoencoder neural network for landslide susceptibility prediction. Landslides. (2020) 17:217–29. 10.1007/s10346-019-01274-9

[58] Anguita D Ghio A Ridella S Sterpi D. K-Fold Cross Validation for Error Rate Estimate in Support Vector Machines. in DMIN. Las Vegas (2009). pp. 291–7.

[59] Kohavi R. A study of cross-validation and bootstrap for accuracy estimation and model selection. in Proceedings of the 14th international joint conference on Artificial intelligence. Montreal, QC (1995). pp. 1137–45.

[60] Wong T-T. Performance evaluation of classification algorithms by k-fold and leave-one-out cross validation. Pattern Recognit. (2015) 48:2839–46. 10.1016/j.patcog.2015.03.009

[61] Ranjan G Verma AK Radhika S. K-nearest neighbors and grid search cv based real time fault monitoring system for industries. in 2019 IEEE 5th International Conference for Convergence in Technology (I2CT). Bombay: IEEE (2019). pp. 1–5.

[62] Ahmed MR Ahammed MS Niu S Zhang Y. Deep Learning Approached Features for ASD Classification using SVM. in 2020 IEEE International Conference on Artificial Intelligence and Information Systems (ICAIIS). Dalian: IEEE (2020). pp. 287–90.

[63] Buitinck L Louppe G Blondel M Pedregosa F Mueller A Grisel O. API design for machine learning software: experiences from the scikit-learn project. In: European Conference on Machine Learning and Principles and Practices of Knowledge Discovery in Databases, Prague workshop: Languages for Data Mining and Machine Learning. arXiv preprint arXiv:13090238 (2013). p. 108–22.

[64] Ng YL Lo MCK Lee KH Xie X Kwong TN Ip M. Development of an open-access and explainable machine learning prediction system to assess the mortality and recurrence risk factors of clostridioides difficile infection patients. Adv Intell Syst. (2021) 3:2000188. 10.1002/aisy.20200018825855820

[65] Bisong E. More supervised machine learning techniques with scikit-learn Building Machine Learning and Deep Learning Models on Google Cloud Platform. Cham: Springer (2019). pp. 287–308.

[66] Kirori Z. Hyper-parameter optimization: toward Convolutional Neur. Res J Comput Inf. (2019) 7:1–5. Available online at: http://www.isca.me/COM_IT_SCI/Archive/v7/i2/1.ISCA-RJCITS-2019-004.pdf

[67] Ho TK. Random decision forests. in Proceedings of 3rd International Conference on Document Analysis and Recognition. Montreal, QC: IEEE (1995). pp. 278–82.

[68] Rätsch G Onoda T Müller KR. An improvement of AdaBoost to avoid overfitting. in Proc of the Int Conf on Neural Information Processing. Kitakyushu: Citeseer (1998).

[69] Hsu C-C Lee Y-C Lu P-E Lu S-S Lai H-T Huang C-C. Social media prediction based on residual learning and random forest. Proceedings of the 25th ACM International Conference on Multimedia. Mountain View, CA: (2017). pp. 1865–70.

[70] Zhang Y Haghani A A. gradient boosting method to improve travel time prediction. Transport Res Part C Emerg Technol. (2015) 58:308–24. 10.1016/j.trc.2015.02.019

[71] Joshi P. Python Machine Learning Cookbook. Birmingham: Packt Publishing Ltd (2016).

[72] Bergstra J Bengio Y. Random search for hyper-parameter optimization. J Mach Learn Res. (2012) 13:281–305. Available online at: https://www.jmlr.org/papers/volume13/bergstra12a/bergstra12a34798506

[73] Appelhans BM Luecken LJ. Heart rate variability as an index of regulated emotional responding. Rev Gen Psychol. (2006) 10:229–40. 10.1037/1089-2680.10.3.229

[74] Wang C Wang F. An emotional analysis method based on heart rate variability. I: *Proceedings of 2012 IEEE-EMBS International Conference on Biomedical and Health Informatics*. Hong Kong: IEEE (2012). pp. 104–7.

[75] Di Simplicio M Costoloni G Western D Hanson B Taggart P Harmer C. Decreased heart rate variability during emotion regulation in subjects at risk for psychopathology. Psychol Med. (2012) 42:1775. 10.1017/S0033291711002479 22067596

[76] Zhu J Ji L Liu C. Heart rate variability monitoring for emotion and disorders of emotion. Physiol Meas. (2019) 40:064004. 10.1088/1361-6579/ab1887 30974428

[77] Kleiger RE Stein PK Bigger Jr JT. Heart rate variability: measurement and clinical utility. Ann Noninvas Electrocardiol. (2005) 10:88–101. 10.1111/j.1542-474X.2005.10101.x 15649244 PMC6932537

[78] Shaffer F McCraty R Zerr CL. A healthy heart is not a metronome: an integrative review of the heart's anatomy and heart rate variability. Front Psychol. (2014) 5:1040. 10.3389/fpsyg.2014.01040 25324790 PMC4179748

[79] Taylor JA Carr DL Myers CW Eckberg DL. Mechanisms underlying very-low-frequency RR-interval oscillations in humans. Circulation. (1998) 98:547–55. 10.1161/01.CIR.98.6.547 9714112

[80] Bloomfield DM Magnano A Bigger Jr JT Rivadeneira H Parides M Steinman RC. Comparison of spontaneous vs. metronome-guided breathing on assessment of vagal modulation using RR variability. Am J Physiol Heart Circ Physiol. (2001) 280:1145–50. 10.1152/ajpheart.2001.280.3.H1145 11179058

[81] Shi H Yang L Zhao L Su Z Mao X Zhang L. Differences of heart rate variability between happiness and sadness emotion states: a pilot study. J Med Biol Eng. (2017) 37:527–39. 10.1007/s40846-017-0238-0

[82] Heathers JA. Everything Hertz: methodological issues in short-term frequency-domain HRV. Front Physiol. (2014) 5:177. 10.3389/fphys.2014.00177 24847279 PMC4019878

[83] Reyes del Paso GA Langewitz W Mulder LJ Van Roon A Duschek S. The utility of low frequency heart rate variability as an index of sympathetic cardiac tone: a review with emphasis on a reanalysis of previous studies. Psychophysiology. (2013) 50:477–87. 10.1111/psyp.12027 23445494

[84] Quintana DS Guastella AJ Outhred T Hickie IB Kemp AH. Heart rate variability is associated with emotion recognition: direct evidence for a relationship between the autonomic nervous system and social cognition. Int J Psychophysiol. (2012) 86:168–72. 10.1016/j.ijpsycho.2012.08.012 22940643

[85] Billman GE. The LF/HF ratio does not accurately measure cardiac sympatho-vagal balance. Front Physiol. (2013) 4:26. 10.3389/fphys.2013.00026 23431279 PMC3576706

[86] H. Ke. Improving Brain E-Health Services via High-Performance EEG Classification With Grouping Bayesian Optimization. IEEE Trans Serv Comput. (2020) 13:696–708. 10.1109/TSC.2019.296267327295638

[87] Ke H Chen D Shah T Liu X Zhang X Zhang L. Cloud-aided online EEG classification system for brain healthcare: a case study of depression evaluation with a lightweight CNN. Softw Pract Exp. (2020) 5:596–610. 10.1002/spe.266825855820

[88] Acharya UR Oh SL Hagiwara Y Tan JH Adam M Gertych A. A deep convolutional neural network model to classify heartbeats. Comput Biol Med. (2017) 89:389–96. 10.1016/j.compbiomed.2017.08.022 28869899

[89] Yildirim Ö. A novel wavelet sequence based on deep bidirectional LSTM network model for ECG signal classification. Comput Biol Med. (2018) 96:189–202. 10.1016/j.compbiomed.2018.03.016 29614430

[90] Erdenebayar U Kim Y Park JU Lee S Lee KJ. Automatic classification of sleep stage from an ecg signal using a gated-recurrent unit. Int J Fuzzy Logic Intell Syst. (2020) 20:181–7. 10.5391/IJFIS.2020.20.3.181

