# Supplementary material for: Heart Rate Information-Based Machine Learning Prediction of Emotions Among Pregnant Women
Source: Front Psychiatry. 2022 Jan 27;12:799029. doi: 10.3389/fpsyt.2021.799029 (PMC8830335; doi:10.3389/fpsyt.2021.799029)
Supplement: Supplementary file 1 [file Data_Sheet_1.docx]

**Supplementary Table 1:** Descriptions and functions of HRV

| Features | Description | Function |
| --- | --- | --- |
| CVRR | Coefficient of variance of RR-intervals | Represents autonomic nervous function |
| SDNN | Standard deviation of the time interval between successive normal heart beats | Represents all the cyclic components responsible for variability in the period of recording and, thus, the total variability of autonomic nervous function |
| RMSSD | Square root of the mean of the sum of the squares of differences between adjacent RR-intervals. Reflects high frequency (fast or parasympathetic) influences on HRV | Represents vagal tone |
| NN50 | Number of interval differences of successive RR-intervals greater than 50 ms | Represents vagal tone |
| pNN50 | Proportion derived by dividing NN50 (number of interval differences of successive RR-intervals greater than 50 ms) by the total number of RR-intervals | Represents vagal tone |
| LF | Low frequency from 0.04 to 0.15 Hz | Represents the activity of sympathetic and parasympathetic nerves |
| HF | High frequency from 0.15 to 0.4 Hz | Represents the activity of the parasympathetic (vagus) nerve |
| LF/HF | The Ratio of LF to HF | Represents the overall balance of the sympathetic and parasympathetic nerves |

The table provides a description of all HRV indicators. Among these, CVRR, SDNN, RMSSD, LF, and HF are important features in this study.

**Supplementary Table 2:** Optimal parameters

| Machine learning algorithm name | Methods | Parameters |
| --- | --- | --- |
| SVM | 5-fold cross-validation  GridSearchCV  RandomizedSearchCV | C, gamma |
| KNN |  | n_neighbors, p |
| LR |  | penalty, class_weight, C, intercept_scaling |
| SGD |  | alpha |
| GBT |  | n_estimators, max_depth, max_features, min_samples_split, min_samples_leaf, subsample, criterion, learning_rate |
| XGB |  | n_estimators, max_depth, learning_rate, subsample, colsample_bytree, min_child_weight, gamma |
| DT |  | max_depth, max_features, min_samples_split, min_samples_leaf, splitter, criterion |
| RF |  | n_estimators, max_depth, max_features, min_samples_split, min_samples_leaf, bootstrap, criterion |
| ANN |  | hidden_layer_sizes, solver, max_iter, verbose, activation, learning_rate, alpha |

SVM, support vector machine; k-NN, k-nearest neighbor; SGD, stochastic gradient descent; LR, logistic regression; DT, decision tree; NB, naïve Bayes; RF, random forest; GBT, gradient boosting trees; XGBoost, extreme gradient boosting; ANN, artificial neural network

**Supplementary Table 3:** Test datasets for each model evaluation index (WEKA, random forest)

| Item | Happy | Anxiety | Sad | Frustrated | Weighted Average |
| --- | --- | --- | --- | --- | --- |
| TP rate | 0.924 | 0.054 | 0.001 | 0.355 | 0.728 |
| FP rate | 0.659 | 0.001 | 0.003 | 0.083 | 0.473 |
| Precision | 0.753 | 0.800 | 0.500 | 0.601 | 0.705 |
| Recall | 0.924 | 0.054 | 0.032 | 0.355 | 0.728 |
| F-measure | 0.830 | 0.101 | 0.001 | 0.446 | 0.689 |
| ROC area | 0.718 | 0.666 | 0.769 | 0.700 | 0.712 |

The table provides model evaluation index data. The results show that the random forest is an appropriate method to predict emotions. ROC; receiver operating characteristic curve

**Supplementary Table 4:** Test datasets for each model evaluation index

| Items | SVM | k-NN | SGD | LR | DT | NB | RF | GBT |
| --- | --- | --- | --- | --- | --- | --- | --- | --- |
| Accuracy | 0.51 | 0.60 | 0.50 | 0.63 | 0.55 | 0.50 | 0.66 | 0.65 |
| Precision | 0.55 | 0.56 | 0.55 | 0.54 | 0.53 | 0.54 | 0.62 | 0.63 |
| Sensitivity | 0.52 | 0.60 | 0.51 | 0.63 | 0.55 | 0.62 | 0.66 | 0.66 |
| F1 score | 0.50 | 0.57 | 0.50 | 0.52 | 0.55 | 0.58 | 0.60 | 0.57 |
| AUC | 0.56 | 0.51 | 0.51 | 0.64 | 0.55 | 0.54 | 0.72 | 0.74 |

The data indicate that the prediction accuracy of the models and that the prediction accuracy of eight methods were >0.70, which included random forest (AUC 0.72) and gradient boosting trees (AUC 0.74). HRV indicators were extracted by us. SVM, support vector machine; k-NN, k-nearest neighbor; SGD, stochastic gradient descent; LR, logistic regression; DT, decision tree; NB, naïve Bayes; RF, random forest; GBT, gradient boosting trees; XGBoost, extreme gradient boosting; ANN, artificial neural network, AUC: area under receiver operating characteristic curve, HRV: heart rate variability

**Supplementary Table 5:** Comparison and analysis of different machine learning algorithms

| Support vector machine (SVM) | Strengths | ① SVM is a learning method for small samples with solid theoretical foundation; ② A few support vectors determine the final result and are not sensitive to outliers; ③ It features excellent generalization ability. |
| --- | --- | --- |
|  | Weaknesses | ① It is difficult to implement large-scale training samples; ② It is difficult to solve multi-classification problems. |
| K-nearest neighbor (KNN) | Strengths | ① The training time complexity is lower than that of support vector machine; ② Compared with Naïve Bayes algorithm, it has no hypothesis for data, high accuracy, and insensitivity to outliers. |
|  | Weaknesses | ① Many calculations are required, especially when there are many characteristic numbers; ② When the sample is unbalanced, the prediction accuracy of rare categories is low; ③ Compared with the decision tree model, the KNN model is less interpretable. |
| Logistic regression (LR) | Strengths | ① The training speed is fast, and the number of calculations is only related to the number of features; ② It is simple and easy to understand, and the interpretability of the model is very good; ③ It is suitable for binary classification problems. |
|  | Weaknesses | ① It is hard to deal with unbalanced data; ② The accuracy is not very high, because the form is very simple, and it is difficult to fit the real distribution of data. |
| Naïve Bayes (NB) | Strengths | ① It has high speed for training and query in large quantity; ② Good performance on small-scale data and is able to handle multiple classification tasks; ③ It is not sensitive to missing data, and the algorithm is relatively simple; ④ It is easy to understand. |
|  | Weaknesses | ① High error rate of classification decision; ② It is sensitive to the expression of input data; ③ A priori probability needs to be calculated; ④ Because the assumption of independence of sample attributes is used, the effect is not good if the sample attributes are related. |
| Stochastic gradient descent (SGD) | Strengths | ① More effective use of information, especially when the information is redundant; ② It has rapid convergence speed; ③ Excellent iteration effect; ④ If the sample size is large, the computational complexity of SGD still has advantages. |
|  | Weaknesses | ① Low accuracy; ② SGD is still unable to achieve linear convergence; ③ A single sample does not represent the trend of the whole sample; ④ It is not easy to implement in parallel. |
| Artificial neural network (ANN) | Strengths | ① The accuracy of classification is high; ② Parallel distributed processing ability is strong; ③ It is very robust and features fault tolerance to noisy neural network. |
|  | Weaknesses | ① Neural network needs many parameters; ② The output results are difficult to explain, which will affect the credibility and acceptability of the results; ③ Learning time is too long, and the purpose of learning can be very difficult to achieve. |
| Decision tree (DT) | Strengths | ① Easy to understand the meaning of decision tree after interpretation and explain; ② In a relatively short period of time, it can produce feasible and good results for large data sources; ③ A decision tree can be constructed for data sets with many attributes; ④ Decision tree can be well extended to large databases, and its size is independent of the size of the database. |
|  | Weaknesses | ① For the data with different sample sizes, the information gain results tend to be those with more numerical values in the decision tree; ② Difficult when dealing with missing data; ③ Overfitting can be a problem; ④ Ignores the correlation between attributes in the dataset. |
| Ensemble learnings  (Random forest (RF), gradient boosting trees (GBT)) | Strengths | ① It is very accurate; ② It can handle high-dimensional data; ③ It flexibly handles all kinds of data; ④ The generalization error rate is low, and the accuracy is high; ⑤ Random forest do not cause the overfitting problems because of randomness; ⑥ This algorithm has good ability to overcome the noise. |
|  | Weaknesses | ① Attributes with more values will have a greater impact; ② Unbalanced data lead to the decline of classification accuracy. |
