# Supplementary material for: Heart Rate Information-Based Machine Learning Prediction of Emotions Among Pregnant Women
Source: Front Psychiatry. 2022 Jan 27;12:799029. doi: 10.3389/fpsyt.2021.799029 (PMC8830335; doi:10.3389/fpsyt.2021.799029)
Supplement: Supplementary file 2 [file Data_Sheet_2.pdf]

Supplementary text 1: HRV

Time domain features were extracted from RR-intervals (RRI).

Python features were extracted. Packages that included pandas were installed.

The RRI format for the analysis first needed to be changed. The function was then set to obtain the time domain features.

The codes are:

```
def get_time_domain_features():
    diff_RRI = np.diff(RRI)
    length_int = len(RRI)
    mean_RRI = np.mean(RRI)
    median_RRI = np.median(RRI)
    range_RRI = max(RRI) - min(RRI)
    RMSSD = np.sqrt(np.mean(diff_RRI ** 2))
    NN50 = sum(np.abs(diff_RRI) > 50)
    pNN50 = 100 * NN50 / length_int
    SDNN = np.std(RRI, ddof=1)
    CVRR = SDNN/ mean_RRI
    Time_domain_features ={}
    return Time_domain_features
```

Next, the frequency domain features were extracted using the following formulae:

$$\widetilde{P_{xx}}(f) = \frac{1}{S}\sum_{t=0}^{S-1} \widetilde{P_{xx}^{(t)}}(f)$$

$$\widetilde{P_{xx}}(f) = \frac{1}{NU}\left|\sum_{m=0}^{N-1} x(m)e^{-i\frac{2\pi km}{N}}\right|^2$$

$$U = \frac{1}{N}\sum_{m=0}^{N-1}|w(m)|^2$$

where, S represents the number of segments, N represents the number of samples, m represents the index of time (0≤m≤N-1), and k represents the frequency index (0≤k≤N-1), and w(m) represents the window function.

(1) Welch’s method was used to estimate the power spectral density of NN intervals

(2) Set parameters

(3) Set index corresponding to LF and HF frequency range

(4) Calculate area under LF and HF curves

(5) Use LF and HF values to calculate LF/HF and LF/(LF+HF)

Supplementary text 2: Machine learning algorithms

1. Support vector machine (SVM)

SVM is defined as a classifier that can be linear or nonlinear and is an example of supervised learning that always focuses on minimizing structural risks (30). There are many parameters in SVM, for example, the regularization parameter. The strength of the regularization is inversely proportional to C, which must be strictly positive. In particular, SVM aims to reduce the number of points with incorrect classification as much as possible, instead of having to classify all points correctly. Thus, noisy data may exist. To a large extent, this method will not make the model too complex and will not cause overfitting. The classification effect is satisfactory for researchers.

Input: training set $\mathbf{T=\{(x_1, y_1), (x2, y2), \ldots, (x_N, y_N)\}, x_i \in R^n, y_i \in \{+1, -1\}, i=1,2,\ldots,N}$

Output: Separating hyperplane and classification decision function.

(1) The convex quadratic programming problem is constructed and solved by selecting a penalty parameter C > 0:

$$\min_{\alpha} \frac{1}{2} \sum_{i=1}^{N} \sum_{j=1}^{N} \alpha_i \alpha_j y_i y_j (x_i \cdot x_j) - \sum_{i=1}^{N} \alpha_i\, i$$

$$s.t.\ \sum_{i=1}^{N} \alpha_i y_i = 0$$

$$0 \le \alpha_i \le C, i = 1,2, \ldots, N$$

to obtain the optimal solution $\mathbf{a^* = (a^*_1, a^*_2, \ldots, a^*_N)^T}$

(2) Calculate:

$$w^* = \sum_{i=1}^{N} \alpha_i^* y_i x_i$$

$$b^* = y_i - \sum_{i=1}^{N} \alpha_i^* y_i (x_i \cdot x_j)$$

(3) Separation hyperplane:

$$\mathbf{w^* \cdot x + b^* = 0}$$

(4) Classification functions:

$$\mathbf{f(x) = sign\,(w^* \cdot x + b^*)}$$

The hyperplane $\mathbf{x = w_0 + w_1 a_1 + w_2 a_2}$ can be written as:

$$\chi = b + \sum_{i\ is\ a\ supp.\ vcctor} \alpha_i\, y_i \vec{a}(i) \cdot \vec{a}$$

2. K-nearest neighbor (KNN)

KNN is a classical, simple, and highly robust classification algorithm. KNN can compare the similarity between testing and training data (16). The KNN algorithm has some advantages compared to other algorithms and is superior to SVM for multiple classification events.

Input: training set T = $(x_1, y_1), (x_2, y_2), \ldots, (x_N, y_N)$

Manhattan distance:

$$L_1(x_i, x_j) = \sum_{l=1}^{n} \left| \chi_i^{(l)} - \chi_j^{(l)} \right|$$

Euclidean distance:

$$L_2(x_i, x_j) = \left(\sum_{l=1}^{n} \left| \chi_i^{(l)} - \chi_j^{(l)} \right|^2 \right)^{\frac{1}{2}}$$

3. Logistic regression (LR)

LR is a machine learning algorithm that analyzes the relationship between predictors. It is commonly used to solve the problem of classification and prediction. For example, LR can distinguish between positive and negative emotions. The algorithm is also used to minimize the error between the result of classification and the value of the label after training for the sample with the result. LR can also construct a separating hyperplane between two datasets (31). LR is a widely used classifier and is particularly suitable for disease prediction.

LR can be calculated using:

$$p(y = c|x) = \frac{exp(w_c^T x)}{\sum_y exp(w_y^T x)'}$$

where, y is an integer index, and weights are encoded into a vector with length K. The features x are re-defined as the result of evaluating the feature functions $f_k(y,x)$ such that there is no difference between the features given by $f_k(y=i,x)$ and $f_k(y=j,x)$. This form is widely used for the last layer in neural network models and is referred to as the softmax function.

4. Random forest (RF)

RF was proposed by Breiman (32). It is a prediction made by synthesizing the prediction results of multiple trees. RF consists of a large number of decision trees (DTs) that choose their splitting features. The trees are built using the classification and regression tree methodology without pruning (32). Prediction is determined by the majority voting of ensemble predictions (33). The many

important parameters include the number of trees in the forest (n_estimators) and the function to measure the quality of a split (criterion). The "criterion" are "gini" for the Gini impurity, "entropy" for the information gain, maximum depth of the tree (max_depth), minimum number of samples required to split an internal node (min_samples_split), and minimum number of samples required to be at a leaf node (min_samples_leaf).

5. Naïve Bayes (NB)

The NB algorithm is a classification algorithm based on Bayes' rule. This algorithm is particularly suitable when the dimensionality of the inputs is high (20). NB is mainly performed through manual data preprocessing to create a dataset that can be used for classifier training and to finally complete the classifier with a specific category classification function.

(1) Classification learning: the probability of the class given an instance

Evidence E = instance's non-class attribute values

Event H = class value of instance

In a naïve assumption, evidence splits into parts (i.e., attributes) that are conditionally independent. This means that given n attributes, Bayes' rule can be written using a product of per-attribute probabilities:

$$\mathbf{P(H|E) = P(E_1|H)P(E_3|H)...P(E_n|H)P(H)/ P(E)}$$

6. DT

DT is a type of classification and prediction model that contains several improvements, especially for software implementation (34). The classification process involves intuitively using probability analysis and searching from top to bottom along a branch down to the leaf node. The label of the leaf node is the final classification category. The DT algorithm controls randomness of the estimator (random_state), which has to be fixed to an integer to obtain a deterministic behavior during fitting. A node will be split if this split induces a decrease in the impurity greater than or equal to this value (min_impurity_decrease), which is the threshold for early stoppage of tree growth. A node will split if its impurity is above the threshold. Otherwise, it is a leaf (min_impurity_split).

The parameter (criterion) is used to determine the calculation method of impurity:

(1) criterion='gini'

$$1 - \sum_{i=0}^{c-1} p(i|t)^2$$

(2) criterion='Entropy'

$$Entropy(t) = -\sum_{i=0}^{c-1} p(i|t)\log_2 p(i|t)$$

where, T represents a given node, I represents any classification of tags, and P(i|t) represents the proportion of label classification I in node t.

7. Gradient boosting trees (GBT)

The GBT iterative DT algorithm can establish a prediction model in the form of an ensemble of weak prediction models. The algorithm has also been used to analyze and classify data (35). The algorithm also involves several parameters. For example, the number of boosting stages to perform (n_estimators) is a function that measures the quality of a split (criterion). The "criterion" is "friedman_mse" for the mean squared error with improvement score by Friedman, "mse" for mean squared error, and "mae" for the mean absolute error, minimum number of samples required to split an internal node (min_samples_split), and minimum number of samples required to be at a leaf node (min_samples_leaf). A node will be split if this split induces a decrease of the impurity greater than or equal to this value (min_impurity_decrease).

8. Stochastic Gradient Descent (SGD)

Machine learning algorithms sometime require a loss function for the original model. The loss function is optimized using an optimization algorithm to identify the optimal parameters and minimize the value of the loss function. SGD is an iterative method to optimize an objective function with suitable smoothness properties. It can be regarded as a stochastic approximation of gradient descent optimization. This reduces the computational burden, especially in high-dimensional optimization problems, achieving faster iterations with a trade-off of a lower convergence rate (36,37).

Gradient descent:

(1) Given a conditional probability model $\mathbf{p(y|x;\ \theta)}$

(2) Parameter vector θ data $\boldsymbol{\tilde{y}_i, \tilde{x}_i, i = 1 \dots N}$

(3) Prior on parameters, $\mathbf{p(\theta;\ \lambda)}$ with hyper-parameter $\boldsymbol{\lambda}$

(4) Gradient descent with learning rate η can be written as:

$$g = \frac{\partial}{\partial \theta}\left[-\sum_{i=1}^{N} \log p(\tilde{y}_i|\tilde{x}_i;\theta) - \log p(\theta;\lambda)\right]$$

$$\theta \leftarrow \theta - \eta g$$

For convex models, the change in the loss or the parameters is often monitored, and the algorithm is terminated when it stabilizes.

9. XGBoost (XGB)

XGB is a scalable tree boosting algorithm and an efficient implementation of the gradient boosting algorithm (38). The basic learners in XGB can be either cart or linear. According to the parameters of XGB, when a node is split, it will be split only if the value of the loss function decreases after splitting. Gamma specifies the minimum loss function descent required for node splitting. The larger the value of this parameter, the more conservative is the algorithm. In general, the operation speed and algorithm accuracy of XGB are better than those of GBT.

10. Artificial neural network (ANN)

Multi-layer perceptron (MLP) is also an ANN. In addition to the input and output layers, multiple hidden layers can exist. The simplest MLP contains only one hidden layer, which is a three-layer structure. An ANN model is a mathematical model or calculation model that imitates the structure and function of a network. The ANN classification algorithm belongs to the supervised machine learning algorithm. ANN is characterized by a pattern of connections between the neuron architecture and determination of the weights of the connections training or learning algorithm (39). ANN has been widely used to analyze and classify data (40,41).

Supplementary text 3: Accuracy, precision, sensitivity, specificity, F1 score, and area under curve (AUC) are defined as follows using the true positive (TP), false positive (FP), false negative (FN), and true negative (TN) summarized in the following table:

		True	
	Total samples	Positive	Negative
Predictive	Positive	TP	FP
	Negative	FN	TN
	TP + FN + FP + TN	Positive samples: TP + FN	Negatives samples: FP + TN

(1) Accuracy = (TP + TN)/(TP + TN + FP + FN)

(2) Precision = TP/(TP + FP)

(3) Sensitivity = Recall = TP/ (TP + FN)

(4) Specificity = TN/ (TN + FP)

(5) F1 score = (2*Precision*Recall)/(Precision + Recall)

Suppose M is the total number of positive samples, and N is the total number of negative samples:

(6) AUC = (sum of positive sample numbers – M*(M + 1)/2)/M*N
