# Supplementary figures and images for: Heart Rate Information-Based Machine Learning Prediction of Emotions Among Pregnant Women
Source: Front Psychiatry. 2022 Jan 27;12:799029. doi: 10.3389/fpsyt.2021.799029 (PMC8830335; doi:10.3389/fpsyt.2021.799029)

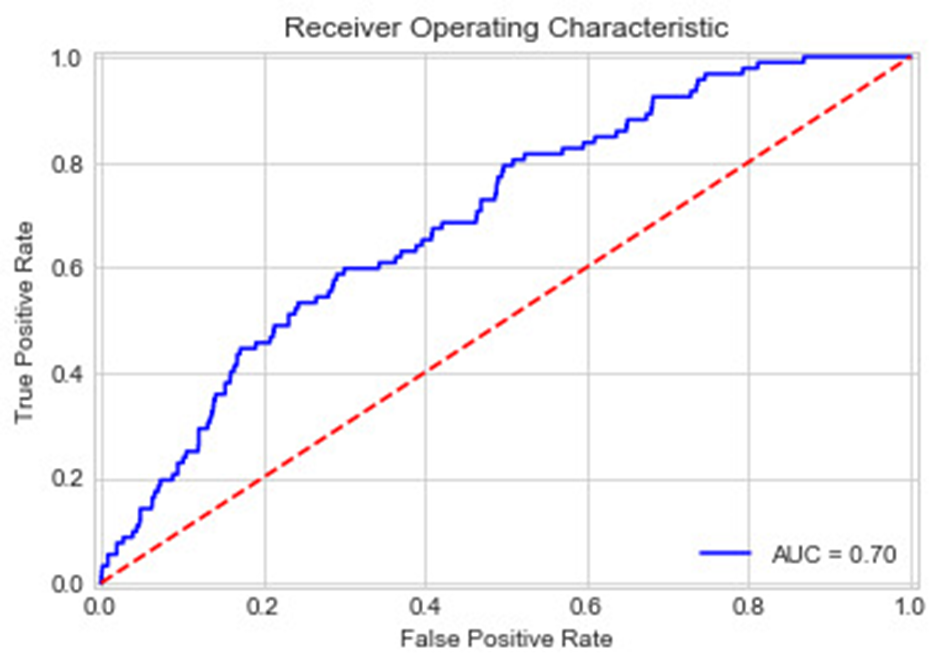

Supplement: Supplementary file 3 [file Image_1.TIF]

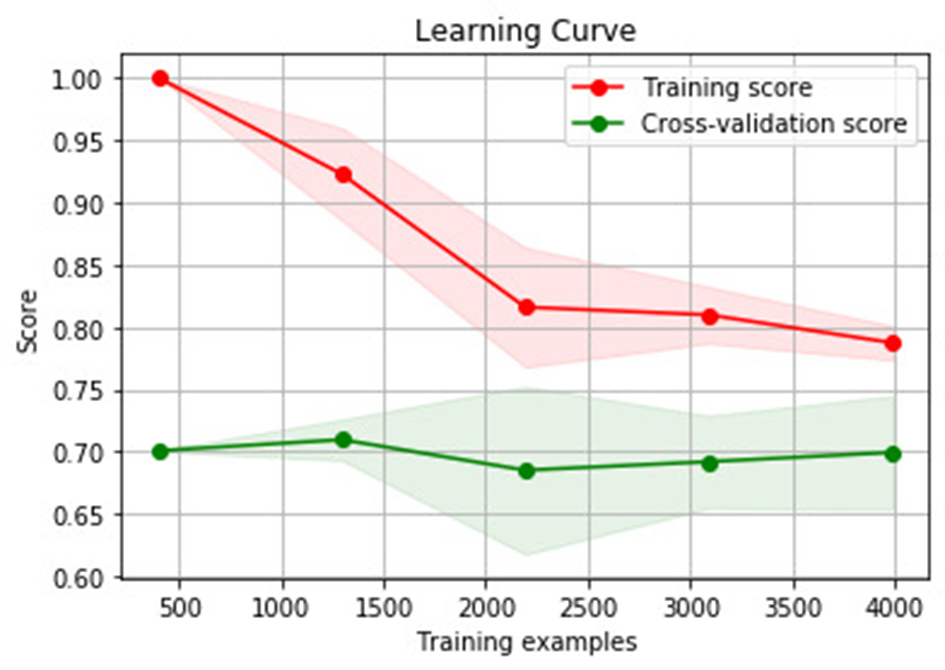

Supplement: Supplementary file 4 [file Image_2.TIF]

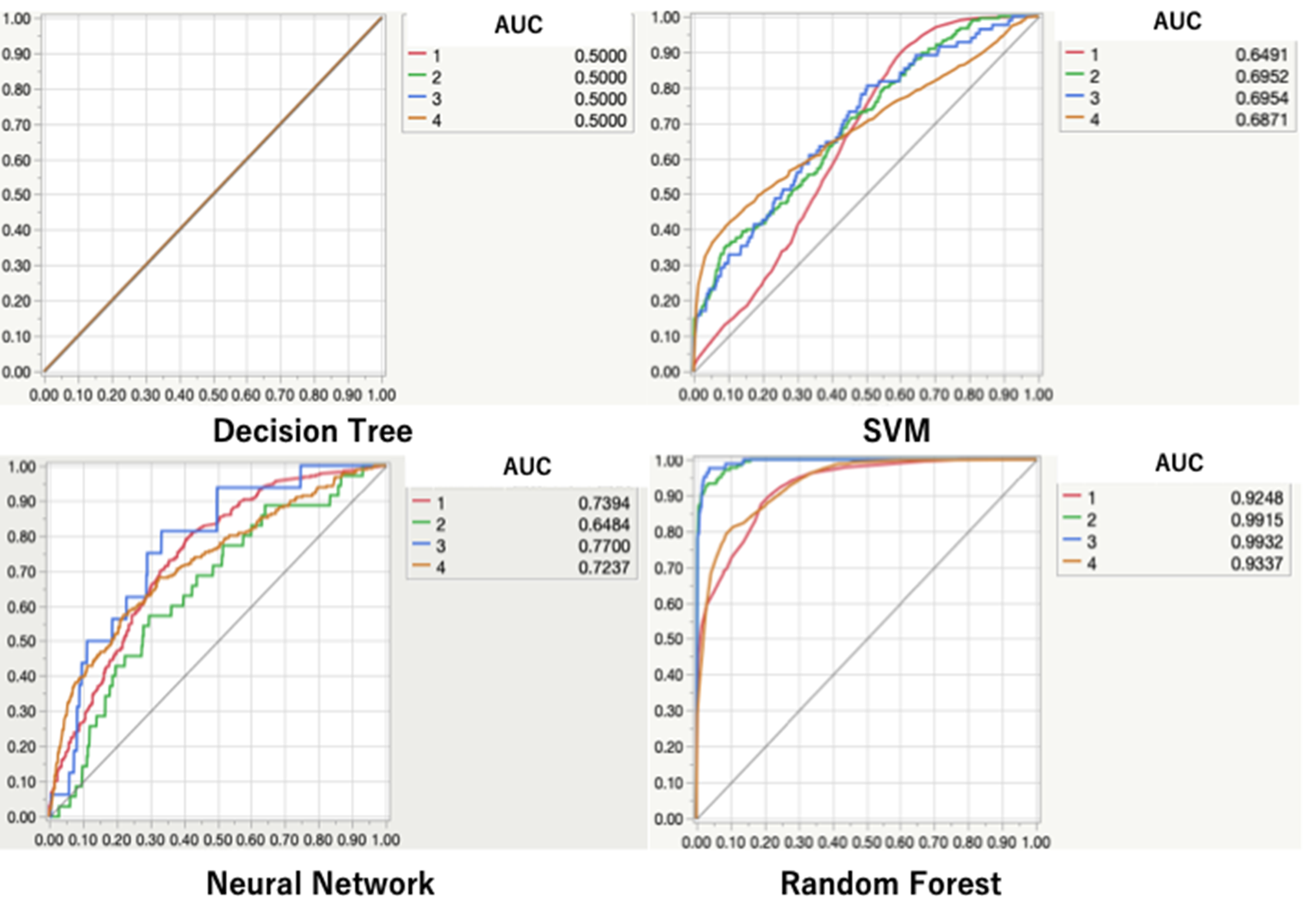

Supplement: Supplementary file 5 [file Image_3.TIF]
